# Does preference for self‐reliance moderate associations of health‐related social control with physical activity and smoking cessation? Two intensive longitudinal studies

**DOI:** 10.1111/bjhp.70082

**Published:** 2026-05-14

**Authors:** Pascal Küng, Walter Bierbauer, Corina Berli, Patrick S. Höhener, Janina Lüscher, Tania Bermudez, Anna Banik, Aleksandra Luszczynska, Urte Scholz

**Affiliations:** ^1^ University of Zurich Zurich Switzerland; ^2^ University of Bern Bern Switzerland; ^3^ Swiss Paraplegic Research Nottwil Switzerland; ^4^ University of Lucerne Lucerne Switzerland; ^5^ St. Gallen University of Teacher Education St. Gallen Switzerland; ^6^ SWPS University Warsaw Poland

**Keywords:** health‐behaviour, persuasion, physical activity, preference for self‐reliance, pressure, smoking cessation, social control

## Abstract

**Objectives:**

This study examined daily associations between experiencing health‐related social control (persuasion and pressure) from a close other and moderate‐to‐vigorous physical activity (MVPA), smoking abstinence, reactance‐related behaviours (oppositional behaviour), and positive and negative affect. Additionally, we investigated whether daily and person‐mean preference for self‐reliance moderated these associations.

**Design:**

A secondary analysis of two 21‐day daily‐diary studies was conducted: one tracked MVPA in patients after cardiac rehabilitation (*n* = 137), and another investigated adults' attempts to quit smoking (*n* = 71).

**Methods:**

Participants completed daily questionnaires. Minutes of MVPA were measured using hip‐worn accelerometers, and smoking abstinence was assessed dichotomously with carbon monoxide monitors. Bayesian multilevel models tested within‐person effects.

**Results:**

Across both studies, higher daily *pressure* was linked to increased odds of above‐average reactance‐related behaviours. Pressure was further unfavourably associated with MVPA and affect in the cardiac rehabilitation sample, but unrelated to smoking abstinence and affect in the smoking cessation study. Daily *persuasion* only showed a small favourable association with positive affect in the smoking cessation sample. However, exploratory moderation analyses indicated it might be more effective for improving health behaviour when coming from a romantic partner. Neither daily nor person‐mean preference for self‐reliance moderated any associations, but the latter displayed unfavourable associations with affect and reactance‐related behaviour in the smoking cessation study.

**Conclusions:**

Daily pressure appears consistently counterproductive. Associations for persuasion may be more nuanced, potentially shaped by factors like the message source. Although preference for self‐reliance did not moderate any associations, its unfavourable direct associations warrant further investigation.

**Statement of contribution**

**What is already known on this subject?**

- Individuals experience health‐related social control from close others involving persuasion and pressure.
- Persuasion is associated with favourable outcomes, while pressure is linked to reactance and unfavourable outcomes.
- Social control shows same‐day associations, but the role of context and moderators remains understudied.

**What does this study add?**

- Daily pressure is consistently related to reactance‐related outcomes across contexts, samples and sources.
- Associations for daily persuasion are more nuanced; when coming from romantic partners, it may be more effective
- Daily and mean self‐reliance do not moderate any associations, but they are linked to unfavourable outcomes.

## INTRODUCTION

Promoting health behaviours and reducing risk behaviours are essential for overall health and well‐being (Stanaway et al., 2018). For instance, both physical inactivity and smoking are major risk factors for non‐communicable diseases such as cardiovascular disease and cancers, and greatly increase all‐cause mortality (Stanaway et al., 2018). Yet, the global prevalence of these risk behaviours remains high: approximately 22% of adults smoke, and about 31% do not meet the recommended physical activity guidelines (World Health Organization, 2023, 2024). Changing health behaviours is challenging: many smokers make 30 or more quit attempts before remaining abstinent (Chaiton et al., 2016). Therefore, identifying factors that facilitate health behaviour adoption and maintenance is crucial.

Growing evidence suggests that close relationships, such as those with friends or romantic partners, strongly impact health behaviours (Pietromonaco & Collins, 2017). The Dyadic Health Influence Model (DHIM; Huelsnitz et al., 2022) outlines several pathways through which these interpersonal processes may operate. One key pathway is health‐related social control, which involves attempts by one individual to influence another's health behaviour (Huelsnitz et al., 2022; Lewis & Rook, 1999). How the target person responds to such attempts, both in their health behaviours and affect, depends on how the control attempt is enacted. Non‐autonomy‐limiting or autonomy‐promoting social control, referred to as positive control or *persuasion*, has been linked to improved health behaviour, more positive affect and less negative affect (Craddock et al., 2015; Huelsnitz et al., 2022). In contrast, autonomy‐limiting strategies, termed negative control or *pressure*, may backfire and are typically associated with unfavourable behavioural and affective outcomes (Craddock et al., 2015).^1^ These associations can be explained by the intertwined model of reactance (Brehm, 1966; Dillard & Shen, 2005). The distinct associations of persuasion and pressure may be explained by appraisals and attributions elicited by respective strategies (Frijda, 2007; Logic et al., 2009). Persuasion may be appraised as helpful and individuals may perceive their partners' motive as stemming from concern or from wanting them to succeed with their health goals (Huelsnitz et al., 2022). Consequently, persuasion may signal care and speak to the need for relatedness (Deci et al., 2017). According to the extended mediational model of social control, these positive appraisals may facilitate positive affect and motivational readiness, which may account for the positive association between persuasion and health behaviour change (Logic et al., 2009). Conversely, pressure may be perceived as a threat to autonomy. According to the intertwined model of reactance (Brehm, 1966; Dillard & Shen, 2005), this perceived threat triggers reactance, a motivational state defined as a combination of a negative affective component (i.e., anger and resentment; Logic et al., 2009) and negative cognitions (e.g., formulating counterarguments). This leads to a more negative attitude towards the message or request and lowers their intention to comply. Whenever reactance results in oppositional behaviours (i.e., smoking or refusing to engage in physical activity), we refer to these as *reactance‐related behaviours*.

While past cross‐sectional research on health‐related social control has led to valuable insights, these designs do not allow for conclusions about how these processes unfold in individuals' everyday lives (Hamaker, 2012; Scholz, 2019). Recently emerging intensive longitudinal studies with daily measures have started to replicate these associations within‐person: persuasion relates to favourable same‐day outcomes, while pressure is linked to reactance and unfavourable outcomes (Küng et al., 2025; Scholz et al., 2021). Furthermore, findings suggest that today's social control does not show significant associations with health behaviour on subsequent days, indicating that underlying processes are fast‐acting (Küng et al., 2025; Scholz et al., 2021).

Although the general associations between health‐related social control and the role of reactance are broadly understood, less is known about how individual differences, contexts and relationship factors moderate these processes—an area identified as a research priority (i.e., Pietromonaco & Collins, 2017). Notably, prior research has primarily focused on romantic couples, whereas other forms of (close) relationships have received less attention. Additionally, factors such as attachment style, relational beliefs and social goals may influence how individuals perceive and respond to social exchange processes (Huelsnitz et al., 2022; Pietromonaco & Collins, 2017). For instance, research shows that some individuals react more strongly to persuasive messages, indicating a higher *trait proneness for reactance* (Dillard & Shen, 2005). Similarly, one study showed that patients who believe spouses should be more involved in their partners' health display less resistance and hostility in response to spousal control attempts (Rook et al., 2011). An inversely related construct is preference for self‐reliance, which is defined as how much individuals prefer to manage challenges alone rather than receiving external help or influence (Snyder, 2017). Although largely unexplored in the context of health‐related social control, this construct has been addressed in the broader social support literature, with studies showing that individuals with a higher preference for self‐reliance benefit less from social support when dealing with stress (Snyder, 2017). It is therefore possible that such individuals may also benefit less from persuasion and react more negatively to pressure, perceiving control attempts as more autonomy‐threatening and against their wishes.

While preference for self‐reliance is often considered a trait, it may also fluctuate within individuals over time and across situations. Previous research has indicated that behaviours and experiences fluctuate from day to day, even in how personality traits are expressed (Wilson et al., 2017). In line with this, the Whole Trait Theory considers traits as density distributions of states, where individuals vary not only in their average levels but also in the extent to which their states fluctuate (i.e., their variance across time and situations) and in the overall shape of their distribution (Fleeson & Jayawickreme, 2015). Therefore, both the stable and fluctuating aspects of preference for self‐reliance may shape how targets respond to daily health‐related social control.

The goal of the current study is to examine the daily associations of experienced social control strategies (persuasion and pressure) from a self‐selected close other with health behaviours, reactance‐related behaviours, positive affect and negative affect, while considering both person‐mean preference for self‐reliance (as a proxy for the underlying stable aspect of the trait), and daily fluctuations from that person‐mean (i.e., state) as moderators. Data were drawn from two intensive longitudinal daily diaries datasets focused on distinct contexts and health behaviours: (1) moderate‐to‐vigorous physical activity (MVPA) among patients after cardiac rehabilitation and (2) smoking cessation in adults from the general population. While hypotheses regarding the two distinct health behaviours were addressed in the respective datasets only, all other hypotheses were separately investigated across both datasets. All hypotheses were preregistered on OSF (https://osf.io/zxay9).

Based on current literature, our hypotheses regarding *persuasion* were:

(Main within‐person effects of persuasion): On days when individuals receive more persuasion than usual from a close other, they (a) engage in more MVPA, (b) are more likely to abstain from smoking, (c) experience more positive affect and (d) experience less negative affect.

(Within‐person moderation): On days when individuals report a higher preference for self‐reliance than usual, the favourable associations of persuasion with (a) MVPA, (b) smoking abstinence, (c) positive affect and (d) negative affect are weaker.

(Cross‐level moderation): For individuals with a higher average preference for self‐reliance than others, the daily favourable associations of persuasion with (a) MVPA, (b) smoking abstinence, (c) positive affect and (d) negative affect are weaker than for persons with a lower average preference for self‐reliance.

Our hypotheses regarding *pressure* were:

(Main within‐person effects of pressure): On days when individuals receive more pressure than usual from a close other, they (a) engage in less MVPA, (b) are less likely to abstain from smoking, (c) display more reactance‐related behaviours, (d) experience less positive affect and (e) experience more negative affect.

(Within‐person moderation): On days when individuals report a higher preference for self‐reliance than usual, the unfavourable associations of pressure with (a) MVPA, (b) smoking abstinence, (c) reactance‐related behaviours, (d) positive affect and (e) negative affect are stronger.

(Cross‐level moderation): For individuals with a higher average preference for self‐reliance than others, the daily unfavourable associations of pressure with (a) MVPA, (b) smoking, (c) reactance‐related behaviours, (d) positive affect and (e) negative affect are stronger than for persons with a lower average preference for self‐reliance.

Additionally, we conducted exploratory moderation analyses to investigate whether daily associations of social control varied by source (romantic partner vs. others).

## MATERIALS AND METHODS

### Sample and design

#### Study 1 – Cardiac rehabilitation

The ‘Cardiac Rehabilitation, Adjustment Disorder, Medication Adherence, and Physical Activity (CAMP)’ project was an observational study investigating patients with cardiac diseases during and after inpatient rehabilitation in Switzerland. At baseline, participants were asked to identify a significant other, who was subsequently referenced in questionnaire items related to social exchange processes. The period relevant to the current analysis occurred upon discharge when participants took part in a 21‐day daily‐diary phase. During this phase, participants completed end‐of‐day questionnaires on study‐provided tablets and wore hip‐worn accelerometers to track their MVPA. The study adhered to ethical standards and was approved by the Cantonal Ethics Committee of Zurich, Switzerland (REQ‐2017‐00508), and all participants provided informed consent. Participants received CHF 130 for completing the study. Further information is accessible on the OSF repository of the original project (https://osf.io/4eqdj).

A total of *n* = 137 patients completed the 21‐day diary phase between April 2018 and July 2019. The participants' mean age was 62.28 years (SD = 11.15), and 85% were male. Sixty‐nine percent were married or in a relationship. On average, participants had a body mass index (BMI) of 26.06 kg/m^2^ (SD = 4.34) and self‐reported engaging in 292.45 min (SD = 153.30) of MVPA per week at baseline. Primary diagnoses included ischaemic heart disease (54%), valve disorders (27%), heart failure (8%) and aortic aneurysm (6%). On average, they took 6.72 medications (SD = 2.13) and had 6.42 concurrent diagnoses (SD = 1.73). As an indicator of socio‐economic status, most participants (57%) had standard health insurance (universal basic coverage), 25% had semi‐private health insurance (higher‐cost supplementary plan) and 18% had private health insurance (highest‐cost supplementary plan).

#### Study 2—Smoking cessation

The ‘Smoking Cessation with Smartphone Applications; SWAPP’ project evaluated the effectiveness of the *SmokeFree Buddy app* in supporting smoking cessation among adults who smoked at least one cigarette per day and intended to quit (Lüscher et al., 2019). Participants were randomly assigned to an intervention group or a control group. Both groups filled out daily questionnaires and used the iCO Smokerlyzer (Bedfont Scientific Limited, 2012), a handheld device connected to a corresponding app, to measure and self‐monitor daily carbon monoxide levels as an indicator of smoking abstinence. The intervention group additionally used the SmokeFree Buddy app and self‐selected a ‘buddy’ from their social network. Each participant was required to select a buddy who was currently a non‐smoker from their social network. The intervention was not effective for promoting smoking abstinence, but it reduced the number of cigarettes smoked in the short term (Schwaninger et al., 2021). Only participants from the intervention group were included in the current analysis, as only they provided data on experienced social control from their buddies. The period relevant to this study was the 21‐day assessment phase, beginning with the participants' self‐set quit date. The trial was approved by the University of Zurich's Institutional Ethics Commission (no. 17.12.13), and all participants provided informed consent. Participants received a lottery entry for a chance to win a grand prize of CHF 200 and 40 vouchers worth CHF 50 each, and they were allowed to keep the Smokerlyzer (valued at CHF 60). Buddies received CHF 50 upon completing the study. Further details can be found in (Lüscher et al., 2019), and the trial's results are presented in (Schwaninger et al., 2021) and (Berli et al., 2025).

A total of *n* = 71 participants from the intervention group contributed data for the 21‐day period after their self‐set quit date. Data were collected between May 2018 and September 2019. The participants' mean age was 31.27 years (SD = 11.21, range = 19–62), and 39% were female. At baseline, 10% smoked 21 cigarettes or more per day, 55% smoked 11–20 cigarettes, and 35% smoked up to 10 cigarettes daily. Forty‐two percent of participants were in a romantic relationship, of which 55% were cohabiting (representing 23% of the total sample). Furthermore, among those in a relationship, 71% reported a smoking partner (representing 30% of the sample). The self‐selected (non‐smoking) buddy was most often a friend (45%), followed by a romantic partner (27%), a colleague (10%), a sibling (7%) and a parent (6%). Regarding education, 18% had completed vocational training, 37% had earned a university degree, and 53% were still in training or retraining. In terms of income, 21% earned between CHF 6001 and CHF 8000 (which encompasses the Swiss median monthly net income of CHF 6538; Bundesamt für Statistik BFS, 2018), while 48% earned less and 31% earned more.

### Measures

Measures were largely equivalent between the two studies, except for the different health behaviours. Health behaviours were assessed device‐based, and all other focal measures with end‐of‐day questionnaires. Table 1 provides a comprehensive description of all focal measures and highlights wording differences between the two studies. Intraclass correlation coefficients (ICCs), along with within‐ and between‐person correlations, are presented in Table 2 (cardiac rehabilitation study) and Table 3 (smoking cessation study).

**TABLE 1:** Items and descriptive statistics of focal variables in the cardiac rehabilitation study and the smoking cessation study.

Construct	Study 1 (cardiac rehabilitation)					Study 2 (smoking cessation)					
	Item	*n*	% missing	M	SD	Item		*n* (%)	% missing	M	SD
Persuasion Adapted from Butterfield and Lewis (2002)	How much did {your close other} try today to influence you through constructive suggestions, recognition, or other positive strategies to engage in heart‐healthy behaviour (e.g., being physically active, taking your medications)? (0 = ‘not at all today’, 5 = ‘very strongly today’)	2557	6%	.48	1.02	How much did your buddy try today to influence your smoking behaviour through constructive suggestions, recognition, or other positive strategies? (0 = ‘not at all today’, 5 = ‘very strongly today’)		1045	30%	.32	.85
Pressure Adapted from Butterfield and Lewis (2002)	‘How much did {your close other} try today to influence you through nagging, reproaches, or other negative strategies to engage in heart‐healthy behaviour (e.g., being physically active, taking your medications)?’ (0 = ‘not at all today’, 5 = ‘very strongly today’)	2557	6%	2.16	1.76	‘How much did your buddy try today to influence your smoking behaviour through nagging, reproaches, or other negative strategies?’ (0 = ‘not at all today’, 5 = ‘very strongly today’)		1045	30%	1.42	1.55
Preference for self‐reliance Adapted from Snyder (2017)	‘Today, I would have preferred that other people stay out of my attempt to engage in heart‐healthy behaviour (e.g., being more physically active, taking my medications).’ (0 = ‘not true at all today’, 5 = ‘completely true today’)	2557	6%	.93	1.51	‘Today, I would have preferred that other people stay out of my attempt to quit smoking.’ (0 = ‘not true at all today’, 5 = ‘completely true today’)		1045	30%	.80	1.40
Health behaviour	Daily minutes of moderate‐to‐vigorous physical activity (MVPA) Measured via hip‐worn GT3X+ accelerometers (ActiGraph, Pensacola, FL, USA) MVPA defined by vector magnitude counts >2690 (Sasaki et al., 2011), aggregated per day. Days with <10 h of wear time were excluded (Choi et al., 2011)	2231	18%	48.61	44.75	Daily abstinence from smoking Measured via iCO Smokerlyzer (Bedfont Scientific Ltd.) Readings ≤6 parts per million (ppm) coded as abstinent (1), >6 ppm coded as lapse (0) (Bedfont Scientific Limited, 2012)	No	154 (10%)	36%		
							Yes	802 (54%)			
Reactance‐related behaviour Adapted from Tucker and Anders (2001)	‘Today, I did exactly the opposite of what {your close other} wanted.’ (0 = ‘does not apply at all today’; 5 = ‘applies exactly today’)	2557	6%	.17	.62	‘Today, I did exactly the opposite of what *others* wanted *regarding my smoking behaviour*.’ (0 = ‘not at all today’; 5 = ‘very frequently today’)		1045	30%	.46	1.08
Positive affect Positive and Negative Affect Schedule (PANAS; Thompson, 2007); I‐PANAS‐SF (Watson et al., 1988; Krohne et al., 1996)	‘Today I felt inspired,’ ‘Today I felt alert,’ ‘Today I felt determined,’ ‘Today I felt *active*,’ ‘Today I felt *attentive*.’ (0 = ‘not at all’, 5 = ‘exactly’) Mean of 5 items; Reliability: ω_within_ = .79 ω_between_ = .92	2563	5%	3.70	.97	‘Today I feel inspired’ ‘Today I feel alert’ ‘Today I feel determined’ ‘Today I feel *enthusiastic*’ ‘Today I felt *excited*’ (0 = ‘not true at all today’, 5 = ‘completely true today’) Mean of 5 items; Reliability: ω_within_ = .79 ω_between_ = .90		1045	30%	2.53	.95
Negative affect Positive and Negative Affect Schedule (PANAS; Thompson, 2007); I‐PANAS‐SF (Watson et al., 1988; Krohne et al., 1996)	‘Today I felt upset’ ‘Today I felt nervous’ ‘Today I felt afraid’ ‘Today I felt *hostile*’ ‘Today I felt *ashamed*’ (0 = ‘not at all’, 5 = ‘exactly’) Mean of 5 items; Reliability: ω_within_ = .56 ω_between_ = .95	2563	5%	.28	.58	‘Today I feel upset’ ‘Today I feel nervous’ ‘Today I feel afraid’ ‘Today I feel *distressed*’ ‘Today I feel *scared*’ (0 = ‘not true at all today’, 5 = ‘completely true today’) Mean of 5 items; Reliability: ω_within_ = .73 ω_between_ = .92		1045	30%	1.40	1.03

*Note*: Items and descriptive statistics for the focal variables in the cardiac rehabilitation study (Study 1; 2712 total diary days from 137 participants) and the smoking cessation study (Study 2; 1701 total diary days from 71 participants).

**TABLE 2:** Cardiac rehabilitation study: within‐ and between‐person correlations and intraclass correlation coefficients (ICCs).

	1	2	3	4	5	6	7
1 Persuasion	[.68]	.18***	−.00	.02	.04*	.00	.02
2 Pressure	.32***	[.44]	.06**	−.05**	.14***	−.03	.09***
3 Preference for self‐reliance	.11	.28***	[.52]	−.02	.13***	−.05**	.08***
4 MVPA	.10	−.03	−.22**	[.54]	−.04*	.22***	−.03
5 Reactance‐related behaviour	.05	.34***	.32***	−.12	[.37]	−.06**	.12***
6 Positive affect	.24**	−.13	−.18*	.27***	−.30***	[.69]	−.14***
7 Negative affect	−.12	.21**	.32***	−.11	.39***	−.55***	[.72]

*Note*: ICCs are displayed in square brackets along the diagonal. Within‐person correlations are above the diagonal, and between‐person correlations are reported below the diagonal. MVPA = moderate to vigorous physical activity (in minutes per day).

**TABLE 3:** Smoking cessation study: within‐ and between‐person correlations and intraclass correlation coefficients (ICCs).

	1	2	3	4	5	6	7
1 Persuasion	[.29]	.21***	.06	−.01	.04	.07*	−.00
2 Pressure	.42***	[.33]	.13***	.01	.20***	.01	−.00
3 Preference for self‐reliance	.41***	.35**	[.48]	−.06	.15***	−.10**	.13***
4 Smoking abstinence	.10	−.09	−.06	[.52]	−.19***	.07*	−.11***
5 Reactance‐related behaviour	.09	.34**	.34**	−.27*	[.46]	−.04	.12***
6 Positive affect	−.04	.13	−.15	.01	.11	[.44]	−.46***
7 Negative affect	.26*	.32**	.38**	.07	.24*	−.05	[.52]

*Note*: ICCs are displayed in square brackets along the diagonal. Within‐person correlations are above the diagonal, and between‐person correlations are reported below the diagonal.

In both studies, the following covariates were included: *time* (0 = first diary day, 1 = last diary day) to account for possible trends across the study (Bolger & Laurenceau, 2013), and a dichotomous variable for the *type of day* (0 = weekday, 1 = weekend) to account for potential variation in individuals' routines. When modelling MVPA in the cardiac rehabilitation study, *accelerometer wear time* was also included as a covariate (Choi et al., 2011).

For additional explorative moderation analyses, the *type of relationship* individuals had with their buddies or close others (i.e., the *source of social control*) was coded dichotomously as romantic partner (1) or others (0; including friends, colleagues, siblings and parents) in the smoking cessation study. In the cardiac rehabilitation study, this data was not available, and we used relationship status as a proxy, based on the assumption that most individuals in a romantic relationship selected their partner as their ‘close other’ (August & Sorkin, 2010).

### Data analysis

All analyses were preregistered on OSF (https://osf.io/zxay9). All hypotheses were analysed separately but identically for each dataset. We used R (v4.3.2; R Core Team, 2023) and RStudio (v2023.12.1; Posit Team, 2023) for all analyses. Predictors and covariates were centred following Bolger and Laurenceau's (2013) recommendations, partitioning variance into stable differences between individuals from daily fluctuations within individuals.

MVPA was coded as missing on days with accelerometer wear time below 10 h, because such data are not considered reliable (Choi et al., 2011). Complete‐case analysis was performed with the assumption of data missing at random (MAR). To assess the plausibility of this assumption, we conducted a comprehensive series of missing data diagnostics (full methods and results are reported in Data S1).

We fitted Bayesian multilevel models using the brms package (Bürkner, 2017) with repeated measures nested in participants and a maximal random effects structure (Barr et al., 2013). We included all predictors and moderators in each model. Although this joint analysis constituted a minor deviation from the preregistered analysis plan, it allowed for the estimation of unique effects while controlling for other predictors, presenting stricter tests for our hypotheses (see Data S2 for further details on this decision).

We applied weakly informative priors centred around null effects. This means we started with the conservative assumption that there were no effects, requiring the data to provide strong evidence to move results away from zero. Full estimation settings and conducted model diagnostics are reported in SM4 and in the full analysis code via the linked GitHub repository (https://osf.io/prsdk/).

The medians of the posterior distributions are reported as robust point estimates that are invariant to transformations such as exponentiation, alongside 95% equal‐tailed intervals (ETI). Inferences about significance are based on the probability of direction (*pd*), which is closely related to the frequentist *p*‐value (*p* = 2 * [1 − *pd*]; Makowski et al., 2019). The threshold of *pd* > .975 applied in this study indicates a 97.5% probability that an effect is strictly positive or negative, which corresponds to a standard two‐tailed *p*‐value of .05.

We employed different model families and link functions to account for the distinct distributions of our outcome variables. For *positive affect*, which followed a normal distribution, we used Gaussian models. The resulting unstandardized coefficients (b) represent the additive change in affect per unit increase in the predictor. For *reactance‐related behaviour* and *negative affect*, the data were highly skewed, with many zero values. We dichotomized the outcomes by classifying values above a participant's average as 1 and values at or below their average as 0. In this way, each participant served as their own baseline, facilitating cross‐study comparability. For these dichotomized outcomes and dichotomous *smoking abstinence*, we used Bernoulli models and report odds ratios (OR). These ORs are interpreted as multiplicative changes in the outcome‐odds per unit increase in the predictor. Finally, for *MVPA*, we used a negative binomial model to accommodate the over‐dispersed count‐data. For this model, we report incidence rate ratios (IRR), interpreted as multiplicative changes in the raw outcome score per unit increase in the predictor.

For the exploratory moderation analyses concerning the dichotomous variable representing the source of social control, cross‐level interactions with daily persuasion and pressure were included alongside the other moderators in all models.

## RESULTS

### Study 1 – Cardiac rehabilitation

All results of the cardiac rehabilitation study are reported in Table 4. The intercept for MVPA was IRR = 31.05 (*pd* >.999, 95% CI = [25.85, 37.06]), indicating that the expected daily MVPA for the average person with all predictors at their reference values (i.e., at the beginning of the study, on a weekday, with person‐average levels of persuasion, pressure and preference for self‐reliance) was 31.05 min. The intercept for dichotomized reactance‐related behaviour was OR = .02 (*pd* > .999, 95% CI = [.01, .04]), meaning that when all predictors are at their reference values, the odds of exhibiting reactance‐related behaviour (i.e., doing the opposite) more so than the person‐mean are .02, corresponding to a probability of approximately 2% (.02/[1 + .02]). Similarly, the intercept for dichotomized negative affect was OR = .28 (*pd* > .999, 95% CI = [.21, .38]), corresponding to a probability of about 22% (.28/[1 + .28]) of experiencing negative affect above the person‐mean. The intercept for (non‐dichotomized) positive affect was *b* = 3.64 (*pd* > .999, 95% CI = [3.51, 3.77]), indicating that on days when all predictors are at their reference values, the expected positive affect is 3.64 (scale range: 0–5).

**TABLE 4:** Bayesian multilevel model estimates for daily outcomes in the cardiac rehabilitation study.

Fixed effects	MVPA			Reactance			Positive affect			Negative affect	
	OR	95% CI		OR	95% CI		*b*	95% CI		OR	95% CI
Intercept	31.05***	[25.85, 37.06]		.02***	[.01, .04]		3.64***	[3.51, 3.77]		.28***	[.21, .38]
Within‐person main effects											
Daily persuasion	1.02	[.98, 1.06]		1.19	[.90, 1.64]		.02	[−.01, .04]		1.00	[.85, 1.17]
Daily pressure	.95*	[.90, .99]		1.83***	[1.36, 2.41]		−.04*	[−.07, −.00]		1.41***	[1.19, 1.67]
Daily pref. self‐reliance	.99	[.96, 1.02]		1.19	[.99, 1.49]		−.02	[−.04, .00]		1.04	[.92, 1.18]
Day	1.17**	[1.06, 1.30]		.49*	[.28, .86]		.16***	[.09, .22]		.48***	[.34, .67]
Weekend (0 = No, 1 = Yes)	.92*	[.86, .98]		1.36	[.94, 1.97]		−.02	[−.06, .03]		.77*	[.60, .97]
Daily wear time	1.00	[1.00, 1.00]									
Within‐person moderation											
Daily persuasion × daily pref. self‐reliance	1.02	[.99, 1.06]		1.03	[.83, 1.31]		.02	[−.00, .05]		1.00	[.88, 1.14]
Daily pressure × daily pref. self‐reliance	.97	[.92, 1.02]		1.05	[.86, 1.28]		.00	[−.04, .03]		1.08	[.92, 1.25]
Cross‐level moderation											
Daily persuasion × mean pref. self‐reliance	1.01	[.97, 1.04]		.89	[.72, 1.09]		.00	[−.02, .03]		1.04	[.92, 1.18]
Daily pressure × mean pref. self‐reliance	.97	[.93, 1.01]		.86	[.70, 1.06]		.02	[−.01, .04]		.88	[.76, 1.02]
Between‐person main effects											
Mean pref. self‐reliance	.84*	[.71, .98]		1.51	[.99, 2.29]		−.07	[−.19, .05]		1.17	[.94, 1.47]
Mean persuasion	1.10	[.97, 1.25]		.83	[.59, 1.17]		.21***	[.12, .30]		.78**	[.65, .94]
Mean pressure	.98	[.76, 1.28]		2.64**	[1.40, 5.09]		−.27**	[−.46, −.08]		1.60**	[1.12, 2.30]
Mean wear time	1.004*	[1.00, 1.00]									

Additional parameters	MVPA			Reactance‐related behaviour			Positive affect			Negative affect	
	Estimate	95% CI		Estimate	95% CI		Estimate	95% CI		Estimate	95% CI
Random effects (standard deviations)											
Intercept	.96	[.85, 1.11]		2.07	[1.63, 2.66]		.75	[.66, .85]		1.22	[1.02, 1.47]
Daily persuasion	.06	[.00, .12]		.37	[.06, .68]		.05	[.01, .09]		.36	[.12, .57]
Daily pressure	.03	[.00, .10]		.45	[.04, .93]		.07	[.01, .12]		.30	[.02, .60]
Daily pref. self‐reliance	.02	[.00, .07]		.12	[.01, .39]		.03	[.00, .06]		.19	[.02, .38]
Daily persuasion × daily pref. self‐reliance	.03	[.00, .08]		.30	[.02, .67]		.06	[.04, .09]		.12	[.01, .35]
Daily pressure × daily pref. self‐reliance	.04	[.00, .12]		.14	[.01, .47]		.04	[.00, .09]		.13	[.01, .40]
Distributional and residual parameters											
Shape	2.03	[1.90, 2.16]									
Sigma							.54	[.52, .55]			

*Note*: MVPA = minutes of moderate‐to‐vigorous physical activity, IRRs = incidence rate ratios (for MVPA, negative binomial model), OR = odds ratios (for reactance‐related behaviour and negative affect, Bernoulli models), and *b* = unstandardized coefficient (for positive affect, Gaussian model). Estimates are the medians of the posterior distributions from Bayesian multilevel models predicting daily minutes of MVPA, the odds of above‐average reactance‐related behaviour or negative affect, and daily levels of positive affect (scale range 0–5). Ninety‐five percent credible intervals (CIs) are equal‐tailed, and inference about effect existence is drawn from the probability of direction (*pd*). Outputs that include correlations between random effects are available upon request.

*pd > .975, **pd > .995, ***pd >.9995.

Our hypotheses regarding persuasion were not supported: Daily persuasion was not significantly related to any outcome (H1.0a‐d), and no interactions with preference for self‐reliance were observed (H1.1a‐d and H1.2a‐d). In contrast, all main effects of pressure were in line with our hypotheses. A one‐unit increase in daily pressure was associated with a 5% decrease in MVPA (H2.0a; IRR = .95, *pd* = .991, 95% CI = [.90, .99]). Additionally, each unit increase in pressure corresponded to an 83% increase in the odds of engaging in reactance‐related behaviour beyond the person‐mean (H2.0c; OR = 1.83, *pd* > .999, 95% CI = [1.36, 2.41]) and a 41% increase in the odds of experiencing negative affect above the person‐mean (H2.0e; OR = 1.41, *pd* > .999, 95% CI = [1.19, 1.67]). Finally, each one‐unit increase in daily pressure was associated with a decrease of .04 units in positive affect (H2.0d; *b* = −.04, *pd* = .988, 95% CI = [−.07, −.00]). No interactions with preference for self‐reliance emerged in this sample, not supporting our moderation hypotheses (H2.1a‐e and H2.2a‐e). In exploratory analyses, preference for self‐reliance did not display significant direct associations with any outcome.

The exploratory moderation analysis using relationship status as a proxy for the source of persuasion (see Table S1) revealed one significant interaction: The IRR point estimate of the association between persuasion and MVPA was 10% larger (more positive) for individuals in a relationship compared with those not in a relationship (IRR = 1.10, *pd* = .984, 95% CI = [1.01, 1.19]). The marginal simple slope for individuals not in a relationship was non‐significant (IRR = .95, *pd* = .909, 95% CI = [.88, 1.02]). Conversely, the marginal simple slope for individuals in a relationship was positive and significant (IRR = 1.04, *pd* = .981, 95% CI = [1.01, 1.09]), indicating that for these individuals, each one‐unit increase in persuasion corresponded to a 4% increase in their MVPA. No other associations were significantly moderated by relationship status.

### Study 2 – Smoking cessation

All results of the smoking cessation study are reported in Table 5. The intercept for smoking abstinence was OR = 37.94 (*pd* > .999, 95% CI = [17.32, 90.85]), indicating that the expected odds of abstinence for the average person with all predictors at their reference values (i.e., at their self‐set quit date marking the start of the study, on a weekday, with person‐average levels of persuasion, pressure and preference for self‐reliance) were 37.94, which is equivalent to a probability of approximately 98% (40.08/[1 + 40.08]). The intercept for dichotomized reactance‐related behaviour was OR = .04 (*pd* > .999, 95% CI = [.02, .09]), indicating that when all predictors are at their reference values, the odds of displaying reactance‐related behaviour above the person‐mean are .04, corresponding to a probability of roughly 4% (.04/[1 + .04]). The intercept for dichotomized negative affect, reflecting the odds of experiencing negative affect above the person‐mean, was OR = 1.30 (*pd* = .981, 95% CI = [1.01, 1.68]), or roughly 57% (1.30/[1 + 1.30]). Finally, the intercept for (non‐dichotomized) positive affect was *b* = 2.44 (*pd* > .999, 95% CI = [2.26, 2.62]), meaning that on days when all predictors are at their reference values, the expected positive affect is 2.44 (scale range: 0–5).

**TABLE 5:** Bayesian multilevel model estimates for daily outcomes in the smoking cessation study.

Fixed effects	Smoking abstinence			Reactance‐related behaviour			Positive affect			Negative affect	
	OR	95% CI		OR	95% CI		*b*	95% CI		OR	95% CI
Intercept	37.94***	[17.32, 90.85]		.04***	[.02, .09]		2.44***	[2.26, 2.62]		1.30*	[1.01, 1.68]
Within‐person main effects											
Daily persuasion	.90	[.69, 1.20]		.95	[.74, 1.23]		.05*	[.01, .09]		.92	[.81, 1.05]
Daily pressure	1.04	[.66, 1.72]		2.54***	[1.65, 4.05]		.02	[−.06, .10]		.99	[.76, 1.29]
Daily pref. self‐reliance	1.03	[.74, 1.50]		1.32	[.90, 1.83]		−.07**	[−.12, −.02]		1.36**	[1.12, 1.70]
Day	.27**	[.12, .62]		1.70	[.78, 3.75]		.18*	[.02, .34]		.35***	[.22, .55]
Weekend (0 = no, 1 = yes)	1.10	[.65, 1.90]		.67	[.39, 1.12]		−.02	[−.13, .08]		.65**	[.48, .88]
Within‐person moderation											
Daily persuasion × daily pref. self‐reliance	1.13	[.82, 1.62]		.87	[.69, 1.06]		.02	[−.02, .06]		1.06	[.93, 1.22]
Daily pressure × daily pref. self‐reliance	.80	[.48, 1.32]		1.23	[.71, 2.38]		−.03	[−.10, .03]		1.04	[.84, 1.33]
Cross‐level moderation											
Daily persuasion × mean pref. self‐reliance	.84	[.64, 1.09]		1.08	[.87, 1.34]		.01	[−.03, .04]		1.04	[.91, 1.18]
Daily pressure × mean pref. self‐reliance	.97	[.67, 1.36]		.99	[.73, 1.45]		−.04	[−.09, .02]		1.14	[.94, 1.41]
Between‐person main effects											
Mean pref. self‐reliance	1.34	[.68, 2.68]		1.49	[.75, 2.96]		−.12	[−.30, .07]		1.07	[.92, 1.25]
Mean persuasion	1.98	[.92, 4.27]		.99	[.45, 2.18]		.02	[−.20, .23]		.91	[.76, 1.09]
Mean pressure	.36	[.11, 1.16]		2.10	[.63, 7.42]		.16	[−.20, .53]		.92	[.67, 1.26]

Additional parameters	Smoking abstinence			Reactance‐related behaviour			Positive affect			Negative affect	
	Estimate	95% CI		Estimate	95% CI		Estimate	95% CI		Estimate	95% CI
Random effects (standard deviations)											
Intercept	2.15	[1.73, 2.65]		2.10	[1.56, 2.88]		.67	[.56, .82]		.08	[.00, .27]
Daily persuasion	.27	[.02, .62]		.17	[.01, .50]		.05	[.00, .11]		.20	[.01, .41]
Daily pressure	.20	[.01, .70]		.40	[.02, 1.20]		.04	[.00, .14]		.29	[.02, .75]
Daily pref. self‐reliance	.21	[.01, .65]		.49	[.05, 1.03]		.05	[.00, .13]		.39	[.07, .70]
Daily persuasion × daily pref. self‐reliance	.42	[.05, .85]		.15	[.01, .51]		.03	[.00, .09]		.13	[.01, .36]
Daily pressure × daily pref. self‐reliance	.39	[.02, 1.04]		.83	[.24, 1.82]		.03	[.00, .12]		.14	[.01, .57]
Distributional and residual parameters											
Sigma							.73	[.69, .76]			

*Note*: IRRs = incidence rate ratios (for MVPA, negative binomial model), OR = odds ratios (for reactance‐related behaviour and negative affect, Bernoulli models), and *b* = unstandardized coefficient (for positive affect, Gaussian model). Estimates are the medians of the posterior distributions from Bayesian multilevel models predicting daily minutes of MVPA, the odds of above‐average reactance‐related behaviour or negative affect, and daily levels of positive affect (scale range 0–5). Ninety‐five percent credible intervals (CIs) are equal‐tailed, and inference about effect existence is drawn from the probability of direction (*pd*). Outputs that include correlations between random effects are available upon request.

*pd >.975, **pd >.995, ***pd >.9995.

Our hypotheses regarding persuasion received little support: daily persuasion was only significantly associated with a slight increase in positive affect (H1.0c; *b* = .05, *pd* = .993, 95% CI = [.01, .09]) and had no significant link to smoking abstinence, reactance‐related behaviour and negative affect (H1.0a, H1.0b and H1.0d). Additionally, no significant interactions with preference for self‐reliance emerged (H1.1a‐d and H1.2a‐d).

Similarly, associations for pressure were only partially in line with our hypotheses. Each one‐unit increase in daily pressure was associated with a 154% increase in the odds of engaging in reactance‐related behaviour above the person‐mean (H2.0c; OR = 2.54, *pd* > .999, 95% CI = [1.65, 4.05]). However, daily pressure was not significantly related to smoking abstinence, positive affect and negative affect (H2.0a, H2.0d and H2.0e). Preference for self‐reliance did not significantly moderate any associations (H2.1a‐e and H2.2a‐e). However, in contrast to the cardiac rehabilitation study, exploratory analyses indicated that preference for self‐reliance displayed some significant main effects: For each one‐unit increase in daily (state) preference for self‐reliance, positive affect was .07 units lower (*b* = −.07, *pd* = .997, 95% CI = [−.12, −.02]) and the odds of experiencing negative affect above the person‐mean were 36% higher (OR = 1.36, *pd* = .999, 95% CI = [1.12, 1.70]).

Exploratory moderation analyses revealed that the type of relationship individuals had with their buddy, that is, the source of social control, significantly moderated the association between persuasion and smoking abstinence (see Table S2): The OR point estimate of the association between persuasion and smoking abstinence was 78% larger (more positive) for individuals whose buddy was their romantic partner versus another relationship partner (OR = 1.78, *pd* = .979, 95% CI = [1.02, 3.19]). However, both marginal simple slopes for romantic partners (OR = 1.42, *pd* = .913, 95% CI = [.85, 2.47]), as well as other relationship types (OR = .80, *pd* = .937, 95% CI = [.61, 1.07]) remained non‐significant. No other associations were significantly moderated by relationship status.

## DISCUSSION

This study examined how persuasion and pressure relate to same‐day behavioural and affective outcomes, and whether daily (state) and person‐mean (a proxy for trait) preference for self‐reliance moderates these associations. We analysed daily‐diary data from two samples to explore whether associations are consistent across health behaviours and context: MVPA in patients after cardiac rehabilitation and smoking cessation in adults attempting to quit.

### Persuasion

In both samples, the anticipated favourable associations of persuasion with behavioural and affective outcomes were largely non‐significant, contrary to prior intensive longitudinal findings (i.e., Scholz et al., 2021). In the cardiac rehabilitation sample, persuasion was unrelated to outcomes, possibly because the social control items referenced both MVPA and medication adherence, diluting MVPA‐specific variance. In the smoking cessation sample, persuasion was only linked to slightly higher positive affect. The null finding for smoking abstinence may reflect a ceiling effect with 98% odds of abstinence for the average person on an average day and 52% of participants never lapsing, limiting the potential for improvement through persuasion. Furthermore, persuasion may sometimes be applied *after* a lapse to prevent further smoking. This benefit would be obscured because these days remain classified as lapse days, and the association would be biased towards a negative relationship.

Furthermore, unlike the cardiac rehabilitation sample where no interventions were present, participants in the smoking cessation study selected a buddy who was assigned an explicit role in supporting the quit attempt. This formalization could have made social control attempts appear more legitimate or expected. However, this is not directly supported by our results, as associations for pressure were similar across both samples and favourable associations for persuasion were largely absent only in the smoking cessation sample. Alternatively, social control from a buddy with an assigned supportive role might be perceived as less authentic (e.g., less driven by genuine concern) compared with naturally occurring interactions. This could make persuasion less efficacious while preserving the unfavourable links of pressure, which would align with the observed patterns. While plausible, it remains likely that the methodological factors discussed earlier (ceiling effects and the timing of control relative to lapses) are a primary driver of these null findings. Future research should compare formalized and ‘naturally occurring’ control to disentangle these possibilities.

Another potential explanation for the lack of direct associations with persuasion is heterogeneity in relationship types. In both samples, our exploratory moderation analyses showed that the association between persuasion and health behaviour was more positive when the source was a romantic partner than another relationship type. Simple slopes for romantic partners were positive in both samples, but reached significance only in patients after cardiac rehabilitation. In the smoking cessation sample, this effect did not quite reach significance, potentially due to the methodological reasons discussed above. Simple slopes for other sources were negative and non‐significant in both studies. These results align with a prior study showing that persuasion benefited only married individuals, who primarily received persuasion from spouses, but not unmarried individuals, who received persuasion from other sources (August & Sorkin, 2010). However, evidence remains mixed: another study found no moderation (Thorpe et al., 2008), although it was potentially underpowered for moderation analysis. Additionally, some cross‐sectional studies have found favourable associations of persuasion from various other sources (Craddock et al., 2015). It is possible that persuasion is especially effective when delivered by romantic partners, whereas benefits from other sources are smaller and harder to detect in our study given sample‐size constraints. There are several reasons why persuasion may be most effective when coming from romantic partners, including increased closeness, greater mutual understanding and more frequent interactions than other types of dyads, which may allow them to better learn how to influence each other effectively and monitor each other more easily (August & Sorkin, 2010; Rook et al., 2011; Seidel et al., 2012). Moreover, persuasion from a romantic partner may be more expected and perceived as legitimate (Berg & Upchurch, 2007). In the cardiac rehabilitation context, legitimacy may be further strengthened by a more salient and immediate health threat (e.g., Huelsnitz et al., 2022; Lewis & Butterfield, 2005).

The consistent null findings for the association between persuasion and affect are in line with another daily‐diary study of romantic couples (Küng et al., 2025). With affect often fluctuating throughout the day, it is plausible that affective responses to social control may dissipate by the time evening questionnaires are completed (Küng et al., 2025). Future research could use measures tied directly to social control (Scholz et al., 2021) or event‐based experience sampling to assess participants' immediate responses to social control.

### Pressure

In both studies, daily pressure was associated with higher odds of above‐average reactance‐related behaviour, consistent with our hypotheses and reactance theory (Brehm, 1966; Dillard & Shen, 2005). In patients after cardiac rehabilitation, pressure was additionally linked to reduced MVPA (likely conservatively, as pressure items combined MVPA and medication adherence), lower positive affect and higher odds of above‐average negative affect, aligning with prior research (Craddock et al., 2015). However, in the smoking cessation sample, no other associations beyond the link with reactance‐related behaviour emerged. Although not in support of our hypotheses, this is consistent with pressure being ineffective: it did *not increase* odds of abstinence yet increased the odds of reactance‐related behaviours. No moderation by the source of pressure (romantic partner vs. others) emerged in either study. Overall, the pattern of results across both studies is consistent with reactance theory (Brehm, 1966; Dillard & Shen, 2005) and prior research (Craddock et al., 2015; Scholz et al., 2021), indicating that pressure may be ineffective and may backfire across health behaviours, samples and its source. The unexpected null findings regarding affect in the smoking cessation sample mirror the ones for persuasion and may be due to the transient nature of affect, as discussed above.

### Preference for self‐reliance

Contrary to our hypotheses, neither daily nor person‐mean levels of preference for self‐reliance moderated any associations in either sample. In both studies, most measurements of this construct were clustered near zero, with some participants always reporting zero. This is reflected in low means and moderate standard deviations, reported in Table 1. The limited variance and restricted range likely reduced our ability to detect moderation, which often has small effect sizes (McClelland & Judd, 1993; Murphy & Russell, 2017). It is also possible that moderations would emerge only at relatively high daily or mean levels of self‐reliance, both of which rarely occurred in our samples. Prior studies have also found low levels with moderate variability (Ishikawa et al., 2023). Future studies should account for this with larger samples.

Alternatively, preference for self‐reliance may not be an essential moderator. This is surprising, given that the seemingly similar construct of *beliefs about appropriate spousal involvement* emerged as a moderator in previous works (Rook et al., 2011; Seidel et al., 2012). A key distinction between these concepts may be that the latter includes the perceived legitimacy of control attempts. Individuals who believe a high level of spousal involvement is appropriate could still prefer to be self‐reliant, but view control attempts as legitimate and well‐meant, which could be more important. Furthermore, a low preference for self‐reliance might not automatically lead to a high preference for partner involvement. Future studies should further clarify these constructs to enhance our understanding of the conditions under which social control can be beneficial.

Finally, in the smoking cessation sample, daily (state) preference for self‐reliance was directly associated with lower positive and higher negative affect. This may indicate that either (1) individuals feel worse on days when they prefer to manage their health alone, or (2) they express a stronger preference for self‐reliance on days when their affect is already diminished. The absence of similar associations in the cardiac rehabilitation sample may be due to greater affective stability in that group. It is possible that the smoking cessation sample was generally more irritable (i.e., experienced more affect fluctuations; Despoina et al., 2017) due to nicotine withdrawal symptoms, which could reinforce any negative associations.

Although preference for self‐reliance did not function as a moderator, these direct associations suggest that it may still be relevant to health behaviour change in other ways. For instance, it may predict decreased support‐seeking behaviour (Ishikawa et al., 2023).

### Strengths and limitations

Our study has several strengths. Examining two distinct samples (patients after cardiac rehabilitation and individuals attempting to quit smoking) allows conclusions about whether associations occur across contexts, enhancing generalizability. The intensive longitudinal designs allow for within‐person analyses, contributing to the emerging literature on daily associations. Furthermore, this design allowed us to operationalize preference for self‐reliance as a density distribution of states (Fleeson & Jayawickreme, 2015), capturing both stable and fluctuating aspects of this trait. This constitutes a major strength that led to increased ecological validity compared with a single static measure, though future studies could employ longer assessment periods to buffer against unusual occurrences that might skew the person‐mean. Another strength was the use of device‐based measures (hip‐worn accelerometers and carbon monoxide monitors), which reduced shared‐method variance and biases inherent to self‐reports. Finally, preregistering our hypotheses and procedures while using an open materials and open code approach ensures transparency and replicability.

Nonetheless, there are notable limitations. Limited variability in reactance‐related behaviour and negative affect necessitated dichotomization, resulting in some loss of information. Combined with the infrequent occurrence of social control and limited variability in preference for self‐reliance, our ability to detect small effects, particularly moderation, was likely diminished. This highlights the need for larger samples or longer assessment periods. Additional measurement limitations included the use of single‐item measures—precluding reliability analyses—and the conflation of MVPA and medication adherence in the cardiac sample's social control item, possibly diminishing associations. Moreover, relationship status had to serve as a proxy in the cardiac sample; however, moderation patterns were consistent with the smoking cessation sample where relationship type was measured directly. Given the exploratory nature of these analyses, future research should replicate them and distinguish between specific relationship types rather than dichotomizing romantic partners versus ‘others’. This necessitates larger samples with mixed dyad types. Additionally, both studies were correlational, precluding causal conclusions. Men were overrepresented in our cardiac (85%) and smoking cessation (61%) samples compared with the broader populations of Swiss cardiac patients (73%; Tessitore et al., 2023) and Swiss people who smoke (55%; Swiss Federal Statistical Office, 2024). However, this composition still mirrors the inherent male predominance within these groups. Consequently, generalizability to the general population might be limited, but our findings remain relevant for these specific populations. Finally, although missing data diagnostics support the MAR assumption (see Data S1), MNAR cannot be fully ruled out. However, any potential bias in the cardiac sample is likely minimal due to low missingness (6%). In the smoking sample, potential systematic missingness on lapse days would likely underestimate pressure's adverse associations (yielding conservative estimates) and overestimate persuasion's favourable links (which appears unproblematic due to our null findings regarding abstinence).

## CONCLUSION

Across two studies, daily pressure was consistently linked to higher odds of above‐average reactance‐related behaviour on the same day. In patients after cardiac rehabilitation, pressure was additionally associated with less same‐day MVPA, while in the smoking cessation sample, it was unrelated to smoking abstinence. This suggests that pressure may backfire regardless of context and individual differences. In contrast, the anticipated favourable associations of persuasion were mostly absent. However, exploratory moderation analyses suggested persuasion may be more positive when coming from a romantic partner, possibly due to greater mutual understanding and the ability to monitor one another's behaviour. Finally, neither daily (state) nor person‐mean (as a proxy for trait) preference for self‐reliance moderated any associations, possibly due to limited variability in our samples (i.e., consistently low values) or because they are not critical moderators. Nonetheless, emerging direct unfavourable associations suggest that this construct may be relevant to health behaviour change. Future research should measure immediate affective responses to social control or employ event‐based experience sampling, leverage larger samples to account for limited variability in social control and preference for self‐reliance, and employ experimental designs for causal inference.

## AUTHOR CONTRIBUTIONS

**Pascal Küng:** Conceptualization; methodology; formal analysis; writing – original draft; writing – review and editing. **Walter Bierbauer:** Data curation; investigation; project administration; writing – review and editing. **Corina Berli:** Writing – review and editing; data curation; investigation; project administration. **Patrick S. Höhener:** Writing – review and editing. **Janina Lüscher:** Writing – review and editing; data curation; investigation; project administration. **Tania Bermudez:** Writing – review and editing; data curation; investigation; project administration. **Anna Banik:** Writing – review and editing. **Aleksandra Luszczynska:** Writing – review and editing; supervision. **Urte Scholz:** Writing – review and editing; conceptualization; funding acquisition; project administration; resources; supervision.

## CONFLICT OF INTEREST STATEMENT

The authors declare that they have no conflict of interest to disclose. The funders had no role in the study design, data collection, analysis, data interpretation or the decision to submit the manuscript for publication.

## Supporting information


Data S1

## Data Availability

The data that support the findings of this study are available upon request.

## Footnotes

^1^
While most literature uses the terms *positive and negative social control*, we prefer *persuasion* and *pressure* (Stephens et al., 2009) for their neutral and descriptive nature.
