# Supplementary material for: Does preference for self‐reliance moderate associations of health‐related social control with physical activity and smoking cessation? Two intensive longitudinal studies
Source: Br J Health Psychol. 2026 May 14;31:e70082. doi: 10.1111/bjhp.70082 (PMC13173323; doi:10.1111/bjhp.70082)
Supplement: Supplementary file 1 — Data S1 [file BJHP-31-0-s001.docx]

# Supplementary Material (SM)

## Table S1

*Cardiac Rehabilitation Study: Exploratory Moderation analysis with Source: Bayesian Multilevel Model Estimates for Daily Outcomes*

| **Fixed Effects** | **MVPA** | |  | **Reactance** | |  | **Positive Affect** | |  | **Negative Affect** | |
| --- | --- | --- | --- | --- | --- | --- | --- | --- | --- | --- | --- |
|  | OR | 95% CI |  | OR | 95% CI |  | *b* | 95% CI |  | OR | 95% CI |
| Intercept | **23.16***** | [16.42, 32.46] |  | **0.02***** | [0.01, 0.05] |  | **3.76***** | [3.51, 4.01] |  | **0.20***** | [0.12, 0.33] |
| *Within-Person Main Effects* | | | | | | | | | | | |
| Daily Persuasion | 0.95 | [0.88, 1.02] |  | 1.30 | [0.86, 2.02] |  | 0.01 | [-0.04, 0.05] |  | 0.98 | [0.73, 1.30] |
| Daily Pressure | 0.96 | [0.87, 1.07] |  | **2.20**** | [1.30, 3.84] |  | -0.02 | [-0.09, 0.06] |  | **1.87***** | [1.31, 2.69] |
| Daily Pref. Self-Reliance | 0.99 | [0.95, 1.02] |  | 1.21 | [0.99, 1.54] |  | -0.02 | [-0.04, 0.00] |  | 1.04 | [0.91, 1.17] |
| Day | **1.16**** | [1.04, 1.28] |  | **0.42**** | [0.23, 0.78] |  | **0.15***** | [0.08, 0.22] |  | **0.48***** | [0.33, 0.68] |
| Weekend (0 = No, 1 = Yes) | **0.92*** | [0.86, 0.98] |  | 1.39 | [0.95, 2.03] |  | -0.02 | [-0.06, 0.03] |  | **0.76*** | [0.60, 0.96] |
| Daily Wear Time | 1.00 | [1.00, 1.00] |  |  |  |  |  |  |  |  |  |
| *Within-Person Moderation* | | | | | | | | | | | |
| Daily Persuasion x Daily Pref. Self-Reliance | 1.02 | [0.98, 1.06] |  | 1.00 | [0.80, 1.27] |  | 0.02 | [-0.01, 0.05] |  | 1.00 | [0.88, 1.15] |
| Daily Pressure x Daily Pref. Self-Reliance | 0.96 | [0.92, 1.01] |  | 1.04 | [0.84, 1.27] |  | 0.00 | [-0.04, 0.03] |  | 1.08 | [0.91, 1.25] |
| *Cross-Level Moderation* | | | | | | | | | | | |
| Daily Persuasion x Mean Pref. Self-Reliance | 1.01 | [0.98, 1.05] |  | 0.88 | [0.71, 1.08] |  | 0.00 | [-0.02, 0.03] |  | 1.03 | [0.91, 1.18] |
| Daily Pressure x Mean Pref. Self-Reliance | 0.97 | [0.93, 1.01] |  | 0.85 | [0.68, 1.06] |  | 0.01 | [-0.02, 0.04] |  | 0.87 | [0.75, 1.01] |
| Daily Persuasion x Source (Romantic Partner) | **1.10*** | [1.01, 1.19] |  | 0.86 | [0.56, 1.38] |  | 0.01 | [-0.04, 0.07] |  | 1.02 | [0.75, 1.41] |
| Daily Pressure x Source (Romantic Partner) | 0.98 | [0.88, 1.10] |  | 0.78 | [0.42, 1.35] |  | -0.03 | [-0.11, 0.05] |  | 0.70 | [0.47, 1.03] |
| *Between-Person Main Effects* | | | | | | | | | | | |
| Mean Pref. Self-Reliance | 0.87 | [0.73, 1.02] |  | **1.55*** | [1.00, 2.37] |  | -0.08 | [-0.20, 0.04] |  | 1.21 | [0.96, 1.53] |
| Mean Persuasion | 1.06 | [0.93, 1.21] |  | 0.86 | [0.60, 1.24] |  | **0.23***** | [0.14, 0.33] |  | **0.75**** | [0.61, 0.90] |
| Mean Pressure | 0.98 | [0.75, 1.27] |  | **2.66**** | [1.40, 5.23] |  | **-0.29**** | [-0.49, -0.08] |  | **1.55*** | [1.07, 2.25] |
| Mean Wear Time | **1.00*** | [1.00, 1.00] |  |  |  |  |  |  |  |  |  |
| Source (Romantic Partner) | **1.50*** | [1.00, 2.26] |  | 1.09 | [0.39, 3.12] |  | -0.19 | [-0.49, 0.11] |  | 1.56 | [0.89, 2.76] |

| **Additional Parameters** | **MVPA** | |  | **Reactance** | |  | **Positive Affect** | |  | **Negative Affect** | |
| --- | --- | --- | --- | --- | --- | --- | --- | --- | --- | --- | --- |
|  | Estimate | 95% CI |  | Estimate | 95% CI |  | Estimate | 95% CI |  | Estimate | 95% CI |
| *Random Effects (Standard Deviations)* | | | | | | | | | | | |
| Intercept | 0.96 | [0.85, 1.11] |  | 2.06 | [1.61, 2.64] |  | 0.75 | [0.66, 0.85] |  | 1.24 | [1.03, 1.49] |
| Daily Persuasion | 0.06 | [0.00, 0.12] |  | 0.37 | [0.05, 0.69] |  | 0.06 | [0.02, 0.09] |  | 0.39 | [0.16, 0.60] |
| Daily Pressure | 0.03 | [0.00, 0.10] |  | 0.50 | [0.06, 0.99] |  | 0.08 | [0.01, 0.13] |  | 0.29 | [0.02, 0.59] |
| Daily Pref. Self-Reliance | 0.02 | [0.00, 0.07] |  | 0.14 | [0.01, 0.44] |  | 0.03 | [0.00, 0.06] |  | 0.19 | [0.01, 0.39] |
| Daily Persuasion x Daily Pref. Self-Reliance | 0.03 | [0.00, 0.09] |  | 0.27 | [0.02, 0.66] |  | 0.06 | [0.04, 0.09] |  | 0.14 | [0.01, 0.37] |
| Daily Pressure x Daily Pref. Self-Reliance | 0.04 | [0.00, 0.12] |  | 0.15 | [0.01, 0.48] |  | 0.04 | [0.00, 0.09] |  | 0.14 | [0.01, 0.44] |
| *Distributional and Residual Parameters* | | | | | | | | | | | |
| Shape | 2.04 | [1.91, 2.17] |  |  |  |  |  |  |  |  |  |
| Sigma |  |  |  |  |  |  | 0.54 | [0.52, 0.55] |  |  |  |

*Note.* The source of social control is dichotomous with “others” as the reference category and “romantic partners” as the other category. IRRs = incidence rate ratios (for MVPA, negative binomial model), OR = odds ratios (for reactance-related behavior and negative affect, Bernoulli models), and *b* = unstandardized coefficient (for positive affect, Gaussian model). Estimates are the medians of the posterior distributions from Bayesian multilevel models predicting daily minutes of MVPA, the odds of above-average reactance-related behavior or negative affect, and daily levels of positive affect (scale range 0-5). Ninety-five percent credible intervals (CIs) are equal-tailed, and inference about effect existence is drawn from the probability of direction (*pd*). Outputs that include correlations between random effects are available upon request.
**pd* > 0.975, ***pd* > 0.995, ****pd* > 0.9995

## Table S2

*Smoking Cessation Study: Exploratory Moderation analysis with Source: Bayesian Multilevel Model Estimates for Daily Outcomes*

| **Fixed Effects** | **Smoking Abstinence** | |  | **Reactance** | |  | **Positive Affect** | |  | **Negative Affect** | |
| --- | --- | --- | --- | --- | --- | --- | --- | --- | --- | --- | --- |
|  | OR | 95% CI |  | OR | 95% CI |  | *b* | 95% CI |  | OR | 95% CI |
| Intercept | **33.92***** | [14.80, 85.22] |  | **0.04***** | [0.01, 0.09] |  | **2.35***** | [2.15, 2.56] |  | 1.27 | [0.97, 1.66] |
| *Within-Person Main Effects* | | | | | | | | | | | |
| Daily Persuasion | 0.80 | [0.61, 1.06] |  | 0.91 | [0.67, 1.23] |  | **0.07**** | [0.02, 0.12] |  | 0.87 | [0.74, 1.01] |
| Daily Pressure | 1.12 | [0.67, 1.93] |  | **2.76***** | [1.65, 4.78] |  | 0.02 | [-0.08, 0.11] |  | 0.93 | [0.67, 1.28] |
| Daily Pref. Self-Reliance | 1.05 | [0.76, 1.53] |  | 1.32 | [0.91, 1.87] |  | **-0.07**** | [-0.12, -0.02] |  | **1.36**** | [1.11, 1.72] |
| Day | **0.27**** | [0.11, 0.61] |  | 1.68 | [0.75, 3.70] |  | **0.18*** | [0.03, 0.34] |  | **0.35***** | [0.22, 0.56] |
| Weekend (0 = No, 1 = Yes) | 1.08 | [0.63, 1.88] |  | 0.66 | [0.38, 1.11] |  | -0.02 | [-0.13, 0.08] |  | **0.64**** | [0.47, 0.87] |
| *Within-Person Moderation* | | | | | | | | | | | |
| Daily Persuasion x Daily Pref. Self-Reliance | 1.13 | [0.83, 1.62] |  | 0.86 | [0.68, 1.06] |  | 0.02 | [-0.02, 0.06] |  | 1.06 | [0.93, 1.23] |
| Daily Pressure x Daily Pref. Self-Reliance | 0.78 | [0.46, 1.29] |  | 1.24 | [0.71, 2.49] |  | -0.03 | [-0.10, 0.04] |  | 1.03 | [0.82, 1.32] |
| *Cross-Level Moderation* | | | | | | | | | | | |
| Daily Persuasion x Mean Pref. Self-Reliance | 0.89 | [0.68, 1.15] |  | 1.10 | [0.87, 1.38] |  | 0.00 | [-0.04, 0.04] |  | 1.06 | [0.93, 1.23] |
| Daily Pressure x Mean Pref. Self-Reliance | 0.95 | [0.65, 1.35] |  | 0.97 | [0.70, 1.46] |  | -0.04 | [-0.09, 0.02] |  | 1.17 | [0.95, 1.46] |
| Daily Persuasion x Source (Romantic Partner) | **1.78*** | [1.02, 3.19] |  | 1.14 | [0.70, 1.85] |  | -0.07 | [-0.16, 0.02] |  | 1.23 | [0.93, 1.66] |
| Daily Pressure x Source (Romantic Partner) | 0.80 | [0.33, 1.98] |  | 0.80 | [0.30, 2.11] |  | 0.00 | [-0.17, 0.17] |  | 1.25 | [0.70, 2.23] |
| *Between-Person Main Effects* | | | | | | | | | | | |
| Mean Pref. Self-Reliance | 1.39 | [0.70, 2.80] |  | 1.50 | [0.73, 3.10] |  | -0.08 | [-0.27, 0.11] |  | 1.09 | [0.93, 1.28] |
| Mean Persuasion | 1.94 | [0.90, 4.23] |  | 0.98 | [0.44, 2.22] |  | 0.01 | [-0.20, 0.23] |  | 0.91 | [0.76, 1.09] |
| Mean Pressure | 0.35 | [0.11, 1.13] |  | 2.14 | [0.60, 7.70] |  | 0.15 | [-0.21, 0.51] |  | 0.91 | [0.66, 1.26] |
| Source (Romantic Partner) | 1.84 | [0.56, 6.16] |  | 0.99 | [0.25, 4.02] |  | 0.33 | [-0.05, 0.71] |  | 1.12 | [0.82, 1.52] |

| **Additional Parameters** | **Smoking Abstinence** | |  | **Reactance** | |  | **Positive Affect** | |  | **Negative Affect** | |
| --- | --- | --- | --- | --- | --- | --- | --- | --- | --- | --- | --- |
|  | Estimate | 95% CI |  | Estimate | 95% CI |  | Estimate | 95% CI |  | Estimate | 95% CI |
| *Random Effects (Standard Deviations)* | | | | | | | | | | | |
| Intercept | 2.16 | [1.74, 2.66] |  | 2.17 | [1.61, 2.99] |  | 0.66 | [0.55, 0.81] |  | 0.08 | [0.00, 0.27] |
| Daily Persuasion | 0.18 | [0.01, 0.55] |  | 0.17 | [0.01, 0.52] |  | 0.05 | [0.00, 0.11] |  | 0.21 | [0.02, 0.42] |
| Daily Pressure | 0.22 | [0.01, 0.72] |  | 0.47 | [0.02, 1.33] |  | 0.04 | [0.00, 0.14] |  | 0.27 | [0.01, 0.76] |
| Daily Pref. Self-Reliance | 0.21 | [0.01, 0.66] |  | 0.52 | [0.06, 1.08] |  | 0.05 | [0.00, 0.13] |  | 0.40 | [0.08, 0.72] |
| Daily Persuasion x Daily Pref. Self-Reliance | 0.40 | [0.04, 0.82] |  | 0.15 | [0.01, 0.53] |  | 0.02 | [0.00, 0.09] |  | 0.14 | [0.01, 0.36] |
| Daily Pressure x Daily Pref. Self-Reliance | 0.39 | [0.02, 1.05] |  | 0.88 | [0.28, 1.89] |  | 0.03 | [0.00, 0.12] |  | 0.14 | [0.01, 0.55] |
| *Distributional and Residual Parameters* | | | | | | | | | | | |
| Shape |  |  |  |  |  |  |  |  |  |  |  |
| Sigma |  |  |  |  |  |  | 0.73 | [0.69, 0.76] |  |  |  |

*Note.* The source of social control is dichotomous with “others” as the reference category and “romantic partners” as the other category. IRRs = incidence rate ratios (for MVPA, negative binomial model), OR = odds ratios (for reactance-related behavior and negative affect, Bernoulli models), and *b* = unstandardized coefficient (for positive affect, Gaussian model). Estimates are the medians of the posterior distributions from Bayesian multilevel models predicting daily minutes of MVPA, the odds of above-average reactance-related behavior or negative affect, and daily levels of positive affect (scale range 0-5). Ninety-five percent credible intervals (CIs) are equal-tailed, and inference about effect existence is drawn from the probability of direction (*pd*). Outputs that include correlations between random effects are available upon request.
**pd* > 0.975, ***pd* > 0.995, ****pd* > 0.9995

## Data S1: Missing Data Diagnostics

To assess the potential for bias due to missing data, we conducted sensitivity analyses examining whether missingness was accounted for by observed variables (MAR) or was plausibly Missing Completely at Random (MCAR) (Little & Rubin, 2019). We focused these diagnostics primarily on the *daily questionnaires* for the following reasons:

1. Accelerometer data (MVPA) in the cardiac sample was missing by design, since it was coded as missing when wear time was below 10 hours. Any influence of latent variables (e.g., low motivation) on data availability operates through wear time. Therefore, by including wear time in the models, the missing data mechanism is fully observed and accounted for (satisfying MAR).
2. The missingness of the CO monitor was almost identical to the missingness of the daily diary (ρ_within_=0.92, *p*<.001). This can be explained by the assessment sequence: participants were prompted to use the iCO-smokerlyzer at the end of the daily diary. Consequently, if participants missed the diary, they did not receive the instructions to complete the CO assessment. Conversely, this also means that they almost always used the CO monitor when prompted. This is an important difference to Study 1, where the accelerometer use was independent from using a smartphone. This dependency also made Intention-to-Treat (ITT) approaches (i.e., "missing equals lapse") infeasible for examining mechanisms, as the necessary diary predictors (e.g., pressure, persuasion) were almost always missing on days when the outcome would be imputed (also see: Blankers et al., 2016; Nelson et al., 2009). For these reasons, our MAR diagnostics focus primarily on modeling the missingness of the daily diary. Due to the high overlap, these findings largely extend to the device-based measure.

A fundamental challenge in analyzing the current data was the presence of *unit non-response*: Across both datasets, there was no within-day partial missingness in self-reported measures; on any given day, either all self-reported variables were recorded, or none were. Consequently, it was not possible to statistically test whether values of one self-reported daily variable (e.g., current affect) were associated with the missingness of other self-reported variables on the same day.

To address this, we employed diagnostic strategies using separate logistic regression models to maximize data usage for each specific test (the complete analysis code and the output from all models is available on OSF under https://osf.io/prsdk:

1. *Cross-Instrument Compliance:* We assessed the relationship between the presence of valid device-based data (accelerometer or CO-monitor) and the completion of the daily diary. This tests whether a common unobserved factor (e.g., low daily compliance or general motivation) was linked to simultaneous non-response.
2. *Autocorrelation:* We tested whether *previous-day* missingness of the diary was associated with missingness on the current day to assess if missingness was linked to routine disruption or unobserved states persisting across days (e.g., low motivation carrying over to the following days). In Study 2, autocorrelation was assessed in a separate model excluding the concurrent device-based predictor due to the very high collinearity between device- and diary-missingness.
3. *Outcome-Dependent Missingness (Same-Day):* In separate models restricted to days where valid device-based data was available, we tested whether the *value* of this behavioral data (e.g., minutes of MVPA or smoking abstinence status) was associated with diary missingness. This examines the possibility that poor behavioral performance (e.g., a lapse in smoking, or low MVPA) led to strategic missingness of the diary (e.g., due to guilt or unwillingness to report the failure).
4. *Lagged Predictors (Within-Domain):* In a third set of models, we tested if self-reported psychological variables from the *previous day* (*t*_−1_) were linked to missingness of the diary or the device-based measures on the subsequent day (*t*_0_). This tests whether negative social interactions or unfavorable affective states may have triggered subsequent attrition (e.g., due to decreased motivation).
5. *Between-Person and Control Predictors:* Finally, we included relevant between-person characteristics (gender, age, BMI, and person-mean levels of pressure, persuasion, and the health behavior) and time variables (diary day, weekend) in the models. This tests whether individuals with specific characteristics (e.g., those with generally lower MVPA) or time trends or specific times were associated with missingness.

**Results**

**Study 1: Cardiac Rehabilitation**

- *Cross-Instrument Compliance:* We found no significant association between providing valid accelerometer data (MVPA) and completing the daily diary on the same day (*OR =* 1.61,95% CI [0.70,3.59]). However, missing accelerometer data on the *previous* day significantly predicted the odds of missing diary data on the following day (*OR =* 2.73,95% CI [1.58,4.64]).
- *Autocorrelation:* We observed significant autocorrelation of missingness (*OR =* 4.42,95% CI [2.56,7.51]), indicating that a missing diary on one day was linked to a significantly increased likelihood of missingness on the subsequent day.
- *Outcome-Dependent Missingness:* On days when valid accelerometer data was available, the *amount* of performed MVPA was unrelated to diary missingness (*OR =* 1.01,95% CI [1.00,1.02]).
- *Lagged Predictors:* Missingness on day *t* was not linked to previous-day pressure (*OR =* 0.88,95% CI [0.70,1.13]), persuasion (*OR =* 0.92,95% CI [0.74,1.15]), reactance-related behavior (*OR =* 1.07,95% CI [0.72,1.72]), positive affect (*OR =* 0.94,95% CI [0.62,1.38]), or negative affect (*OR =* 0.77,95% CI [0.43,1.45]).
- *Between-Person and Control Predictors:* Men were significantly more likely to provide diary data than women (*OR =* 2.58,95% CI [1.04,6.40]). Additionally, a higher BMI was associated with a slight decrease in the likelihood of diary completion (*OR =* 0.91,95% CI [0.84,0.99]). The person-mean level of MVPA did not predict missingness (*OR =* 1.00,95% CI [0.99,1.02]). Neither the study day (*OR =* 0.84,95% CI [0.43,1.68]) nor the presence of a weekend (*OR =* 0.76,95% CI [0.49,1.22]) were related to missingness.

*Conclusion:* Missingness of the daily diary in the cardiac sample was primarily explained by the observed history of missingness (autocorrelation), prior device usage, and demographic factors (Gender, BMI). The finding that missingness may be driven by observed, stable between-person characteristics is consistent with a Missing at Random (MAR) assumption. Because these demographic factors are constant for each individual, they do not confound the estimation of the within-person associations (daily slopes) that are the primary focus of this study.

Theoretically, the autocorrelation we found could reflect routine disruption or latent psychological states persisting across days (e.g., low motivation). However, if states like low motivation were the primary driver of missingness, we might expect (1) lower device-based MVPA on those days, and/or (2) missing accelerometer data (low wear compliance) on those days. Since neither the missingness of MVPA data nor the actual minutes of MVPA (on days with data) was linked to diary missingness, a systematic bias related to such latent states or to poor performance (e.g., hiding inactivity) appears unlikely. The potential impact of any bias related to missingness is further limited by the low rate of missing diaries (6%).

Regarding the device-based measure, as discussed above, MVPA data was Missing at Random (MAR) by design, and including wear time as a covariate in all models with MVPA, we satisfy MAR assumptions.

Taken together, these analyses suggest that the MAR assumption is highly plausible and that any potential bias due to MNAR mechanisms would likely be negligible.

**Study 2: Smoking Cessation**

- *Cross-Instrument Compliance:* Contrary to Study 1, providing valid CO-monitor data was strongly linked to diary completion. Within-person Spearman correlations revealed a very strong association (ρ_within_=0.92, *p*<.001), indicating that on days participants completed the diary, they almost always also used the CO-monitor. Between-person correlations were also high (ρ_between_=0.86,*p*<.001).
- *Autocorrelation:* When tested in a separate model (excluding the same-day CO-monitor usage due to the high collinearity), we observed significant autocorrelation of missingness of the diary (*OR =* 2.09,95% CI [1.43,3.08]), indicating that missing one diary day increased the odds of missing the next.
- *Outcome-Dependent Missingness:* On days when device-based data was available, its value (abstinence vs. lapse) was not linked to diary completion (*OR =* 0.97,95% CI [0.07,12.73]).
- *Lagged Predictors:* Missingness on day *t* was not associated with previous-day levels of pressure (*OR =* 0.80,95% CI [0.58,1.12]), persuasion (*OR =* 1.05,95% CI [0.89,1.25]), reactance-related behavior (*OR =* 0.93,95% CI [0.68,1.26]), positive affect (*OR =* 0.93,95% CI [0.66,1.30]), negative affect (*OR =* 1.36,95% CI [0.98,1.96]), or the previous day’s smoking abstinence status (*OR =* 1.50,95% CI [0.65,3.48]).
- *Between-Person and Control Predictors:* We observed a significant time trend, where the likelihood of diary completion decreased over the course of the study (*OR =* 0.12,95% CI [0.07,0.23]), possibly indicating some study fatigue. We also observed that participants were significantly more likely to complete the diary on weekends (*OR =* 91.51,95% CI [29.12,434.92]). Age, gender and person-mean levels of persuasion, pressure, and abstinence success did not significantly predict missingness.

*Conclusion:* On days where device-based data was available, we found no evidence that having a lapse may deter participants from completing the diary. However, because non-response was largely simultaneous and autocorrelated, we cannot rule out that days with *both* missing device and diary data were characterized by low general motivation, lapses, and potentially less effective persuasion or more detrimental pressure.

Nevertheless, if such days are systematically missing from the analysis, the potential bias would likely operate in the direction of making persuasion appear more effective (by omitting failed persuasion attempts) and pressure seem less harmful (by omitting instances where pressure provoked reactance-related behavior, e.g. smoking or smoking more). Given that we already observed null associations for persuasion regarding smoking abstinence and reactance, and adverse associations for pressure, it is unlikely that missing data has masked true beneficial effects of persuasion or led to a false positive finding regarding the harms of pressure.

To conclude, we argue that MAR remains plausible in the smoking cessation sample, although MNAR may also be plausible. However, given the likely nature of the bias and our patterns of our main results, we believe that our conclusions remain valid.

## Data S2: Model Fitting and Diagnostic Details

**Model Fitting Workflow**

In the preregistration, we planned separate models: one with persuasion as focal predictor and its moderators while controlling only for pressure main effects, and one with pressure as focal predictor and its moderators while controlling only for persuasion main effects. We chose this strategy because we anticipated that estimating all focal effects and moderators jointly could be too complex when retaining a maximal random-effects structure.

We first implemented these preregistered (simpler) models in a frequentist framework using lme4 (Bates et al., 2009). However, even these simpler models did not converge with maximal random effects.

Following the preregistered fallback strategy, we therefore switched to Bayesian multilevel modeling in brms (Bürkner, 2017). This approach is computationally more stable for this level of model complexity and allowed us to estimate the full fixed-effects structure, including all focal predictors, moderators, and planned interaction terms, while retaining participant-level random effects.

While this joint analysis is a minor deviation from the preregistration, this approach estimates unique effects, reducing the risk that shared variance between moderators is misattributed to single terms, and keeps coefficient interpretation comparable across outcomes and studies. It also presents a stricter inferential test because each effect is evaluated conditional on the other terms.

**Estimation**

We used weakly informative priors centered around zero for fixed effects. Intercept, random-effect, and distributional priors were specified by outcome family (e.g., Gaussian, Bernoulli, negative binomial) in the analysis scripts and informed by the empirical means of the response variables.

Main models were estimated with a CmdStan backend using four chains, 2,000 warm-up iterations, and 10,000 sampling draws per chain.

**Diagnostic Workflow**

We used diagnostic metrics (R-hat, estimated Bayesian fraction of missing information, and effective sample size) and visual inspection of the chains to confirm that sampling had stabilized and converged on a consistent solution. As part of this step, we also screened standard Hamiltonian Monte Carlo diagnostics (e.g., divergent transitions and tree-depth warnings).

We then evaluated model adequacy using multiple posterior predictive checks (including empirical cumulative distribution function overlays and scatter-average plots, with log-scale checks for skewed outcomes where useful). Additionally, we used leave-one-out diagnostics to inspect Pareto-k values and identify potentially influential observations.

Finally, we used simulation-based residual diagnostics with DHARMa (Hartig, 2022), including tests for dispersion, zero inflation, and outliers, to assess whether model assumptions were adequately met for each outcome family.

Full reproducible code for estimation settings, diagnostics, and package versions is available in the full analysis scripts available on OSF through the linked GitHub repository (https://osf.io/prsdk).

# References

Bates, D., Maechler, M., Bolker, B., Walker, S., Christensen, R. H. B., Singmann, H., Dai, B., Scheipl, F., Grothendieck, G., Green, P., & others. (2009). Package ‘lme4’. *URL Http://Lme4. r-Forge. r-Project. Org*.

Blankers, M., Smit, E. S., van der Pol, P., de Vries, H., Hoving, C., & van Laar, M. (2016). The Missing=Smoking Assumption: A Fallacy in Internet-Based Smoking Cessation Trials? *Nicotine & Tobacco Research*, *18*(1), 25–33. https://doi.org/10.1093/ntr/ntv055

Bürkner, P.-C. (2017). brms: An R package for Bayesian multilevel models using Stan. *Journal of Statistical Software*, *80*, 1–28.

Hartig, F. (2022). *DHARMa: Residual diagnostics for hierarchical (multi-level / mixed) regression models* (Version 0.4.6) [Computer software]. http://florianhartig.github.io/DHARMa/

Little, R. J. A., & Rubin, D. B. (2019). *Statistical Analysis with Missing Data*. John Wiley & Sons.

Nelson, D. B., Partin, M. R., Fu, S. S., Joseph, A. M., & An, L. C. (2009). Why assigning ongoing tobacco use is not necessarily a conservative approach to handling missing tobacco cessation outcomes. *Nicotine & Tobacco Research*, *11*(1), 77–83. https://doi.org/10.1093/ntr/ntn013
